# Effects of sheep follicular fluid-derived exosomes and miR-148a on theca cell function in vitro

**DOI:** 10.1186/s40659-026-00668-z

**Published:** 2026-01-13

**Authors:** Kai Liu, Runqing Chi, Runan Zhang, Qing Liu, Feng Xing, Yufang Liu, Mingxing Chu

**Affiliations:** 1 State Key Laboratory of Animal Biotech Breeding, Institute of Animal Science, Chinese Academy of Agricultural Sciences, No. 2 Yuanmingyuan West Rd, Beijing, 100193 China; 2 College of Animal Science and Technology, Tarim University, Alar, 843300 Xinjiang China; 3 Key Laboratory of Tarim Animal Husbandry Science and Technology, Xinjiang Production & Construction Corps, Alar, 843300 Xinjiang China

**Keywords:** Sheep, Exosomes, Theca cells, miR-148a, TGFβ2

## Abstract

**Background:**

Exosomes facilitate intercellular communication by transporting proteins, nucleic acids, and other biomolecules, impacting recipient cell functions. In follicular development, exosomes from follicular fluid, secreted by granulosa cells, oocytes, and theca cells (TCs), are essential for follicle health. TCs are key in this process, influencing both development and hormonal output. However, the impact of sheep follicular fluid exosomes on TCs proliferation and the roles of the miRNAs they carry remain unclear. This study aims to investigate these effects, examining how exosomes and their miRNAs influence TCs development and hormone secretion through high-throughput sequencing.

**Methods:**

In this study, exosomes were isolated from sheep follicular fluid by ultracentrifugation, and their integrity was confirmed by determining the particle size distribution by nanoparticle tracking analysis (NTA) and detecting marker proteins such as CD63 and TSG101 by Western blot. Subsequently, an in vitro isolation and culture system for sheep theca cells (TCs) was established, and the cells were treated with 200 µg/mL of exosomes. The functional effects of exosomes were assessed by EdU proliferation assay and ELISA for steroid hormone secretion. The exosome small RNAs were extracted and sequenced, and the 20 miRNAs with the highest expression abundance were screened, and the target genes were predicted using TargetScan8.0 and miRDB, and the predicted genes were analysed by GO and KEGG enrichment. For miR-148a, its mimic and inhibitor were synthesised and transfected into TCs to verify its effects on cell proliferation and hormone secretion; the targeting relationship between miR-148a and transforming growth factor β2 (TGFβ2) was verified by combining with a dual luciferase reporter system, and TGFβ2 was further knocked down by siRNA to evaluate its role in the proliferation of TCs.

**Results:**

The results showed that exosomes with a particle size distribution of 30–150 nm were successfully obtained by ultracentrifugation, which expressed CD63 and TSG101 with good integrity. Screening of exosome concentration showed that 200 µg/mL of exosomes significantly increased the proliferation rate and the secretion level of steroid hormones in TCs. Small RNA sequencing results showed that 130 miRNAs were identified, and the top 20 high-abundance miRNAs predicted 37,343 target genes. GO and KEGG analyses showed that these target genes were significantly enriched in follicle development-related signalling pathways, such as PI3K-AKT, MAPK, Rap1 and Ras. Functional experiments demonstrated that miR-148a mimics could significantly promote TCs proliferation, but had no significant effect on steroid hormone secretion. Dual luciferase and siRNA experiments showed that miR-148a directly targeted the 3’UTR of TGFβ2. Knock-down of TGFβ2 by transfecting its siRNA significantly enhances the proliferation rate of TCs, indicating that miR-148a promotes TC proliferation by down-regulating TGFβ2.

**Conclusions:**

The above findings indicate that sheep follicular fluid exosomes can significantly enhance follicular membrane cell proliferation and steroid hormone secretion. The miR-148a carried in exosomes can promote the proliferation of TCs by inhibiting the expression of its target gene TGFβ2. This study will help to reveal the molecular mechanism of exosomes in follicular development and provide new perspectives for improving reproductive efficiency and genetic improvement in sheep.

**Graphical abstract:**

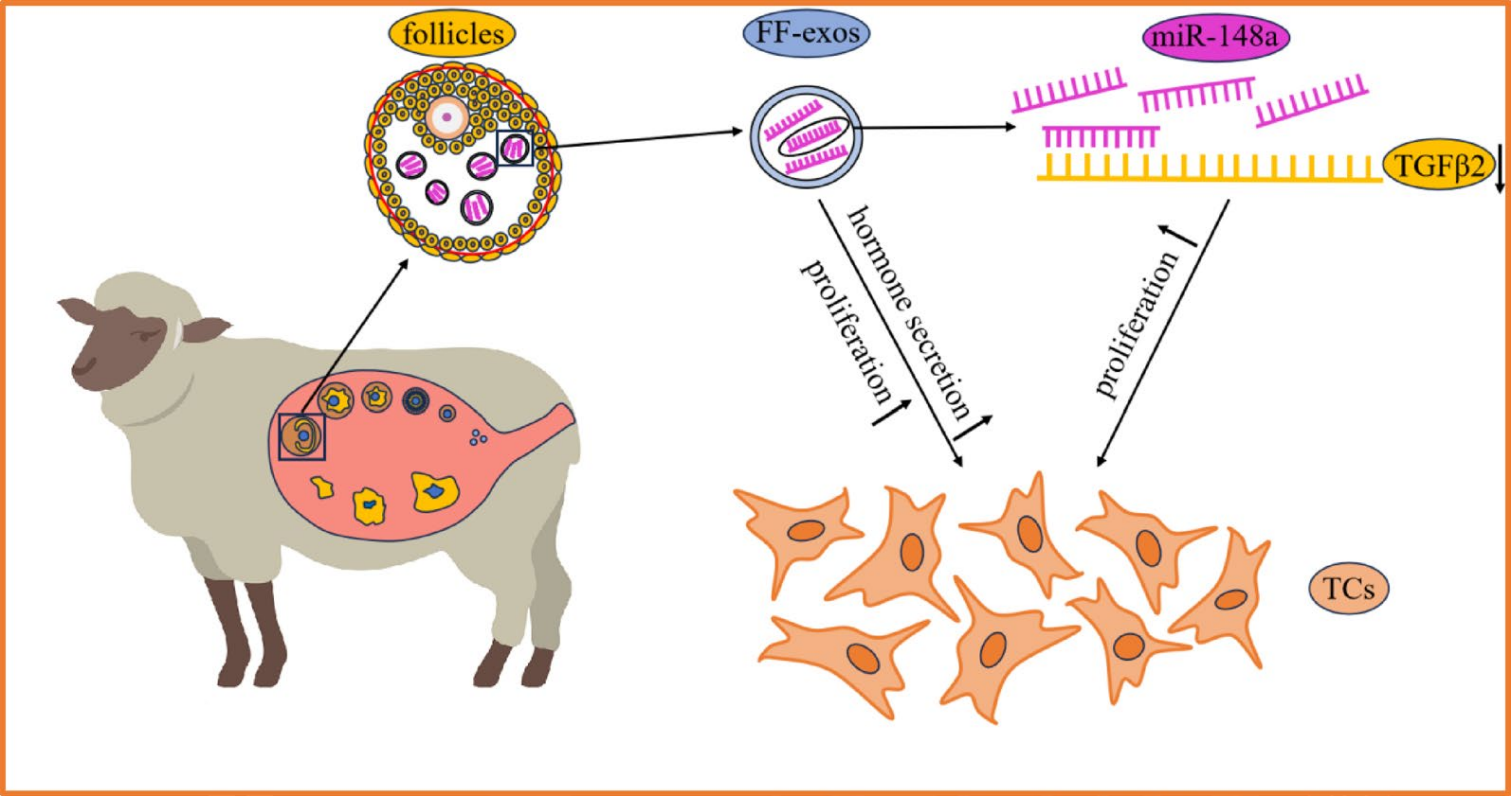

## Background

The follicle is a critical ovarian structure, and its proper maturation determinant of reproductive success [1, 2]. During growth, oocytes, granulosa cells and theca cells (TCs) interact through autocrine and paracrine signals to build the follicular architecture that supports oocyte maturation [3, 4]. TCs act both as the structural scaffold of the follicle and as the primary source of androgens in female mammals; they initially form a supportive layer, then proliferate and differentiate to create a vascular network essential for follicular expansion [5, 6]. Beyond synthesizing progesterone, TCs produce androgens—principally androstenedione and testosterone—that are transported to granulosa cells for oestrogen biosynthesis [5, 7]. However, because TCs are more distant from the oocyte than granulosa cells, their precise roles and developmental mechanisms remain less understood and warrant further investigation. Follicular development, maturation and ovulation are complex, multi-factorial processes [8], and the endocrine activity of TCs is indispensable for normal follicle growth. Enhancing TCs function therefore represents a promising avenue to improve both reproductive performance and economic efficiency in animals.

In recent years, exosomes have garnered significant scientific interest for their role in intercellular communication [9]. These nano-sized vesicles, measuring approximately 30–150 nm in diameter, are secreted by various cell types and are capable of transporting biologically active molecules such as proteins, lipids and miRNAs. These molecules function as autocrine, paracrine or endocrine signals, facilitating cell-to-cell communication and regulating a myriad of biological processes [10–12]. Among these, miRNAs have been particularly scrutinized for their influence on gene expression [13]. Exosomes are particularly enriched in miRNAs, which are small non-coding RNA molecules that operate as cytoplasmic post-transcriptional repressors, modulating mRNA translation into proteins, as well as nuclear transcriptional gene activators [14]. Follicular fluid is a complex mixture composed of circulating plasma at the blood-follicular barrier, secretory products from granulosa cells, oocytes and TCs. It contains a large number of hormones, anti-apoptotic factors, proteins, amino acids and nucleotides, ensuring intercellular communication and providing an important microenvironment for oocyte development and maturation [15, 16]. Since the initial discovery of exosomes in equine follicular fluid in 2012, numerous studies have confirmed the presence of miRNA-laden exosomes in the follicular fluid of various mammals, including pigs, cows, goats and humans [17–21]. Studies have demonstrated that bovine follicular fluid exosomes can enhance ovarian cortical stromal cell proliferation and the synthesis of androstenedione and progesterone [22]. Additionally, bovine and porcine follicular fluid exosomes have been shown to regulate granulosa cell proliferation and hormone synthesis [18, 23]. Furthermore, these exosomes contribute to oocyte development in bovine and porcine [24, 25]. Porcine follicular fluid exosomes also modulate resistance to oxidative stress, proliferation and steroid synthesis in porcine TCs [26]. These discoveries reveal a novel mechanism of intercellular communication within the follicle. However, the impact and mechanisms of exosomes on TCs in sheep remain reported in any study.

In this study, we found that follicular fluid exosomes can regulate the proliferation and steroid hormone secretion of TCs. We identified the miRNA expression profiles of follicular fluid exosomes in sheep and explored the possible mechanisms involved, and found that miR-148a regulates the proliferation of TCs. Thus, these findings provide new insights into improving the efficiency of follicular development and optimizing reproductive technologies in sheep.

## Materials and methods

### Collection of ovaries and cell culture

The Science Research Department (in charge of animal welfare issues) of the Institute of Animal Science, Chinese Academy of Agricultural Sciences (IAS-CAAS; Beijing, P. R. China) has approved all procedures involving laboratory animals. Ethics approval was also granted by the animal ethics committee of IAS-CAAS (No. IAS2020-82). All ovarian tissues were collected post-mortem from a local abattoir. No live animals were used or subjected to any procedures for the purpose of this study.

The ovaries were collected from healthy non-pregnant adult Small Tail Han sheep at a local abattoir. The ewes were of reproductive age and in the estrous cycle. One hundred pairs of sheep ovaries were collected from a local abattoir, placed in a thermos containing 37 °C saline and returned to the laboratory within 2 h. The ovarian tissue was rinsed three times with 75% ethanol and saline, and then placed into a beaker filled with saline. TCs were isolated according to the method of Ma et al. [6]. Use 50 ovaries to isolate TCs cells. The ovary is halved longitudinally and the follicles with a diameter of 3–5 mm (antral follicles) as described by Armstrong et al. [27] were removed. After tearing off as much of the follicular theca externa as possible, the follicle was incised. The inner wall of the inner theca was scraped with a scalpel to remove the mural granulosa cells. The basement membrane is removed as far as possible from the inside of the theca interna using forceps. After rinsing the theca interna three times with PBS, the theca interna was minced by surgical scissors, transferred to a centrifuge tube and digested with 1% IV collagenase at 37 °C for 1 h. The digest was filtered through a 70 µL cell strainer. The filtrate was centrifuged at 4000 ×g for 5 min and the cell precipitate was washed 3 times by resuspension in serum-free medium. The cell sediment was resuspended in DMEM/F12 medium with 10% FBS and inoculated into culture dishes. The cells were incubated in a 5% CO_2_ incubator at 37 °C with saturated humidity, with liquid changes every 24 h, until apposition was complete and used for subsequent experiments.

## Exosomes isolation and purification

The ovarian tissue was rinsed three times in succession with 75% ethanol and saline and then placed in a beaker of saline. Follicular fluid was aspirated from follicles approximately 3–5 mm in diameter using a 5 mL syringe and the collected follicular fluid was collected in a centrifuge tube. To generate a homogenous pool of samples, for every twenty-five ovary follicular fluid samples were mixed as one pooled sample. The follicular fluid obtained was separated from the exosomes by ultracentrifugation according with Maria et al. [28]. The fluid was then centrifuged at 3000 ×g for 30 min at 4 °C to remove macromolecules and cellular debris. Collect the supernatant and centrifuge at 12,000 × g for 30 min at 4 °C. Collect the supernatant in an ultracentrifuge tube and centrifuge at 120,000 × g for 90 min at 4 °C. Discard the supernatant. The precipitate is then resuspended the in PBS and filter through a 0.22 μm membrane. The suspension was centrifuged at 120,000 × g for 90 min at 4 °C, the supernatant was discarded and the precipitate was dissolved in 200 µL of PBS. The concentration of exosomes was determined using the BCA Protein Concentration Kit, and the measured exosomes could be used directly in experiments or dispensed and frozen at −80 °C in a refrigerator for subsequent experiments.

## Transmission electron microscopy (TEM)

Taken 20 µL of the suspension and addedd it to the copper mesh of transmission electron microscope in the form of droplets, let it stand for 1 min, and then negatively stain it with 2% phosphotungstic acid solution for 1–10 min according to Welsh et al. (2024) [29]. Aspirated the excess staining solution, dry it naturally at room temperature (22 ± 2 °C), and then observed it under the bio-type transmission electron microscope and take photographs. Three replicates of each group were repeated three times.

## Nanoparticle tracking analysis (NTA)

Dilute the exosome suspension appropriately to within the detectable range, a final concentration between 1 × 10^8^−1 × 10^9^/mL is generally recommended according to the manufacturer’s instructions [30]. Diluted samples are injected into the NTA instrument and the size and number distribution of exosome particles is determined by laser scattering. Care must be taken during the measurement to ensure that the sample is evenly distributed and that a sufficient number of data points are obtained in a reasonable period of time. Finally, the measured data was analyzed and processed using NTA software to generate particle size and number distribution curves and calculate parameters such as mean particle diameter and most probable number of particles. Three replicates of each group were repeated three times.

## PKH26 exosome uptake test

PKH26 is a dye that fluorescently labels exosomes by binding to lipid molecules in membrane structures. Exosomes were treated with PKH26 (Solarbio, Beijing, China) according to the manufacturer’s instructions (PBS was used as a negative control). PKH26-labelled exosomes were obtained by centrifugation at 120,000 × g for 90 min at 4 °C, and TCs were treated with 200 µg/mL PKH26-labelled exosomes and incubated at 37 °C for 24 h. Cell nuclei were stained with DAPI (Beyotime, China) and cells were observed by confocal microscopy (Nikon, Japan). Three replicates of each group were repeated three times.

### Immunofluorescence staining

TCs were inoculated into 6-well plates and identified by immunofluorescence when the cell density reached 70–80%, fixed in immunofluorescence fixative for 30 min at room temperature (22 ± 2 °C), followed by the addition of immunofluorescence permeabilizing solution for 30 min at room temperature (22 ± 2 °C). Immunostaining blocking solution was added and blocked for 1 h at room temperature (22 ± 2 °C); cells were incubated with rabbit monoclonal antibody vimentin at a concentration of 1:200 or cytokeratin (Bioss, Beijing, China) at 4 °C overnight. The next day, the cells were incubated with rabbit secondary antibody at 1:1000 for 1 h under light protection. The cells were stained with DAPI for 10 min under light protection. The cells were washed three times with PBS at the beginning and end of the staining and between each step. Finally, the images were examined by laser confocal microscopy. Three replicates of each group were repeated three times.

## Cell counting Kit-8 (CCK-8) assay

TCs were cultured in 96-well plates with 100 µL cell culture medium at a density of 1–5 × 10^4^ cells per well. After treatment with different concentrations of exosomes for 24 h, 10 µL CCK-8 solution was added to each well and incubated at 37 °C for 2 h in the dark. The optical density was measured at 450 nm using an enzyme marker. Three replicates of each group were repeated three times.

## Small RNA sequencing and data analysis

Total RNA was extracted from follicular fluid exosome and tested for concentration and purity. A total of four duplicate samples. The construction of sRNA libraries was undertaken in accordance with the manufacturer’s instructions, employing the Illumina TruSeq Small RNA Sample Preparation Kit. After this, the libraries were subjected to quality control analysis using an Agilent 2100 Bioanalyzer system, with the Agilent High Sensitivity DNA Kit being utilized for this purpose. Subsequently, library preparations were subjected to sequencing on the Illumina platform.

Initially, the raw data underwent de-jointing and quality filtering. Subsequent to this, the sequences that passed the filter were de-duplicated (i.e., identical sequences were merged and the corresponding abundance of the sequences was recorded). Subsequently, the de-weighted small RNA sequences were annotated based on the sheep reference genome, and the corresponding annotated abundance was recorded. With the focus on the analysis of microRNAs, the characterization and statistical analysis of microRNA expression were included. Following the statistical analysis, the miRanda software was employed to predict target genes using the 3’UTR sequences of the mRNAs of the species as target sequences. The predicted target genes were then enriched using topGO.

### Quantitative reverse transcription polymerase chain reaction (RT-qPCR)

Total RNA was isolated from the samples using Tissue/Cell RNA Extraction Kit (Tiangen, Beijing, China), and reverse transcription (RT) was carried out utilizing the Reverse Transcription Kit (Tiangen, Beijing, China). The RT‒qPCR analysis was conducted with the SYBR qPCR Master Mix (Roche, Mannheim, Germany), and the reactions were run on a Roche Light Cycler^®^ 480 II system. In these analyses, the *GAPDH* and *U6* were used as endogenous controls to normalize the expression levels of the genes of interest. Relative expression levels were calculated using the 2^−△△Ct^ method [31]. The sequences for the RT-qPCR primers were shown in Supporting Information Tables 1, and these primers were synthesized by Sangon (Shanghai, China). The raw Cq values for GAPDH across all samples ranged from 18.4 to 19.7 (CV = 2.1%), confirming its stable expression under experimental conditions. Specificity was validated by a single-peak melting curve (Tm ± 0.3 °C) and 2% agarose gel electrophoresis (single band at expected size). Three biological replicates of each group were repeated three times.

**Table 1:** Primers used in this study

Gene	Accession number	Primer sequence (5’→3’)	Product length (bp)
*CDK4*	XM_012158548.4	F: GCTGCTGCTGGAGATGCTGAC R: CTCTGCGTCACCTTCTGCCTTG	100
*PCNA*	XM_004014340.5	F: GGCGTCTCAGGCGTTCGTAATC R: CAATGACACGGCTACAGGTACAAGG	104
*CYP11A1*	XM_060401375.1	F: TTTCGCTTTGCCTTTGAGTCCATC R: GCCTCGGTGTCCACTGTGTC	83
*CYP17A1*	NM_001009483.1	F: CTTACCATTGACAAAGGCACAGAC R: GCTTAATGATGGCGAGATGAGTTG	141
*TGFβ2*	XM_004013602.5	F: AGCGACGAGGAATACTACGC R: CTGGGCAGAGAGTTTCCGA	79
*GAPDH*	XM_060411593.1	F: CGGCACAGTCAAGGCAGAGAAC R: CACGTACTCAGCACCAGCATCAC	115
miR-148a	NR_107946.1	F: CGGCGCTCAGTGCACTACAGAACTTTGT	
U6	NR_138085.1	F: CAAGGATGACACGCAATTCG	

### Western blotting

The treated cells were collected when the cell density reached approximately 90–95% and the original medium was discarded. The cells were washed twice with pre-cooled PBS and the configured cell lysate (10 µL each of phosphatase inhibitor, protease inhibitor and PMSF in 1 ml of pre-cooled lysate) was added to the cell sediment (Solarbio, Beijing, China). Cells were lysed on ice for 30 min and then centrifuged at 12,000 rpm for 30 min at 4 °C. Protein concentration was determined using the BCA kit (Solarbio, Beijing, China), BCA working solution was prepared at a 50:1 ratio, then 10 µL of BSA standard was taken and diluted to 100 µL with PBS solution to give a final standard concentration of 0.5 mg/mL. 50 µg protein was sampled. The standard and protein samples were added to the sample wells of a 96-well plate. 200 µL BCA working solution was added to all wells and allowed to stand at 37 °C for 30 min. The absorbance of each well was measured at 562 nm using an enzyme counter. The membrane was separated by electrophoresis using 10% SDS-PAGE and then electrotransferred to a polyvinylidene difluoride membrane. The membranes were blocked in blocking solution for 1 h at room temperature (22 ± 2 °C) and then co-incubated with primary antibody overnight. Following the washing step, the membranes were then subjected to an incubation with a secondary antibody for a period of two hours. Thereafter, western blots were visualized on an Odyssey CLX imaging system (Li-COR) (Bio-Rad, Hercules, CA, USA) with Supersignal HRP chemiluminescent substrate (Beyotime, Beijing, China), in accordance with the manufacturer’s instructions. The signals were quantified using Image J (https://imagej.net/ij/index.html). The primary antibodies used were as follows: CD9 (1:1000; Proteintech, Wuhan, China), CD63 (1:500; Proteintech, Wuhan, China), CD81 (1:500; Bioss, Beijing, China), TSG101 (1:2000; Proteintech, Wuhan, China), CDK4 (1:1000; Proteintech, Wuhan, China), PCNA (1:5000; Proteintech, Wuhan, China), CYP11A1 (1:1000; Proteintech, Wuhan, China), CYP17A1 (1:2000; Proteintech, Wuhan, China), TGFβ2 (1:500; Proteintech, Wuhan, China)and GAPDH (1:50000; Proteintech, Wuhan, China). All primary antibodies used in this study were raised in rabbits. Three biological replicates were utilized for each treatment.

### Enzyme-linked immunosorbent assay (ELISA)

The cell culture medium was collected in a centrifuge tube, and the dead cells were removed via centrifugation. The concentrations of progesterone and androstenedione in the supernatant of the cell culture were determined using an ELISA kit (Meimian, Jiangsu, China). According to the kit specifications, an Epoch microplate reader (Biotech, Winooski, USA) was used to measure the absorbance of 50 µL of the supernatant at 450 nm. Linear regression with the standard curve was then performed, and the corresponding sample concentrations were calculated. The average coefficient of variation between batches was less than 15%, and the progesterone test kit is 1–48 ng/mL, and the sensitivity of the androstenedione test kit is 0.1–12 nmol/L. It is worth noting that during the initial characterization of exosome effects, steroid hormone concentrations are reported per volume of culture medium and were not normalized to cell number or protein content. Three replicates of each group were repeated three times.

### 5-Ethynyl-20-deoxyuridine (EdU) incorporation assay

The EdU assay was performed to assess the proliferation of cells by using a Cell-Light EdU DNA Cell Proliferation Kit (Beyotime, Shanghai, China) following the manufacturer’s protocol. TCs were cultured for 24 h in 24-well plates. TCs were fixed using 4% paraformaldehyde after incubation with 50 mM EdU solution for 2 h. Then, cell lines were sealed with Apollo Dye Solution and Hoechst33342 in sequence. The EdU Cells were observed by confocal microscopy (Nikon, Japan). Three replicates of each group were repeated three times.

### Cell cycle assay

TCs were collected after transfection for 36 h. After washing twice with cold PBS, the cells were fixed with 70% ethanol at 4 °C overnight and re-washed twice to remove any ethanol. Then, we added 100 µL RNase A at 37 °C for 30 min. The cells were then stained with 400 µL propidium iodide (Beyotime, Shanghai, China) for 30 min. FACS was applied to evaluate the cells at 488 nm, and ModFit LT software (Verity Software House) was used for analysis. Three replicates of each group were repeated three times.

### Plasmid construction and transient transfection

The wild-type (WT) and mutant (MUT) 3’-UTR of the *TGFβ2* (GenePharma, Shanghai, China), were successfully cloned into the *SacI/XhoI* sites of the GP-miRGLO vector. SiRNA targeting TGFβ2, miR-148a mimic, miR-148a inhibitor, and their corresponding negative controls were designed and synthesized by GenePharma (Shanghai, China). TCs in good condition were inoculated into 6-well plates and incubated overnight. They were then replaced with 1.5 mL of OPTI-MEM for 30 min. Next, 8 µL of the plasmid to be transfected and Lipofectamine 2000 (Invitrogen, USA) were each diluted in 250 µL of OPTI-MEM to create solutions A and B, respectively. These solutions were mixed well at room temperature (22 ± 2 °C) for 20 min and added to the 6-well plates. Six hours later, the medium was replaced with complete medium for subsequent experiments. Three replicates of each group were repeated three times.

### Dual luciferase activity assay

WT-TGFβ2 (or MUT-TGFβ2) and miR-148a mimics were then co-transfected into HEK293T using Lipofectamine^®^ 3000 (11668019, Invitrogen, USA) according to the manufacturer’s instructions. HEK293T cells were inoculated into 12-well plates and incubated overnight. They were then replaced with 500 µL of OPTI-MEM for 30 min. Next, 4 µL of the plasmid to be transfected and Lipofectamine 3000 (Invitrogen, USA) were each diluted in 50 µL of OPTI-MEM to create solutions A and B, respectively. These solutions were mixed well at room temperature (22 ± 2 °C) for 20 min and added to the 24-well plates. Six hours later, the medium was replaced with complete medium for subsequent experiments. Forty-eight hours after transfection, luciferase activity was measured using a dual luciferase reporter gene assay system (Promega, Madison, USA) according to the manufacturer’s instructions. Three replicates of each group were repeated three times.

### Statistical analysis

Three or more biological replicates were used for statistical analysis in all experiments. The data were analyzed and plotted using GraphPad Prism 9.0 software. The specific statistical test applied to each dataset (e.g., unpaired two-tailed Student’s t-test for comparisons between two groups; one-way ANOVA for comparisons across more than two groups). The assessment of homogeneity of variances was performed using Levene’s test. For data that did not meet the assumption of homogeneity (the steroid hormone concentration data from ELISA), a log10 transformation was applied prior to analysis. All other data met the assumptions of normality and homoscedasticity without transformation. For all one-way ANOVA analyses, Tukey’s honest significant difference (HSD) post-hoc test was used for multiple comparisons. *P* > 0.05 meant no significant difference, *P* < 0.05 meant significant difference, and *P* < 0.01 meant extremely significant difference (ns *P* > 0.05, * *P* < 0.05, ** *P* < 0.01, *** *P* < 0.001).

## Results

### Isolation and identification of follicular membrane cells and follicular fluid exosomes

**Fig. 1:**
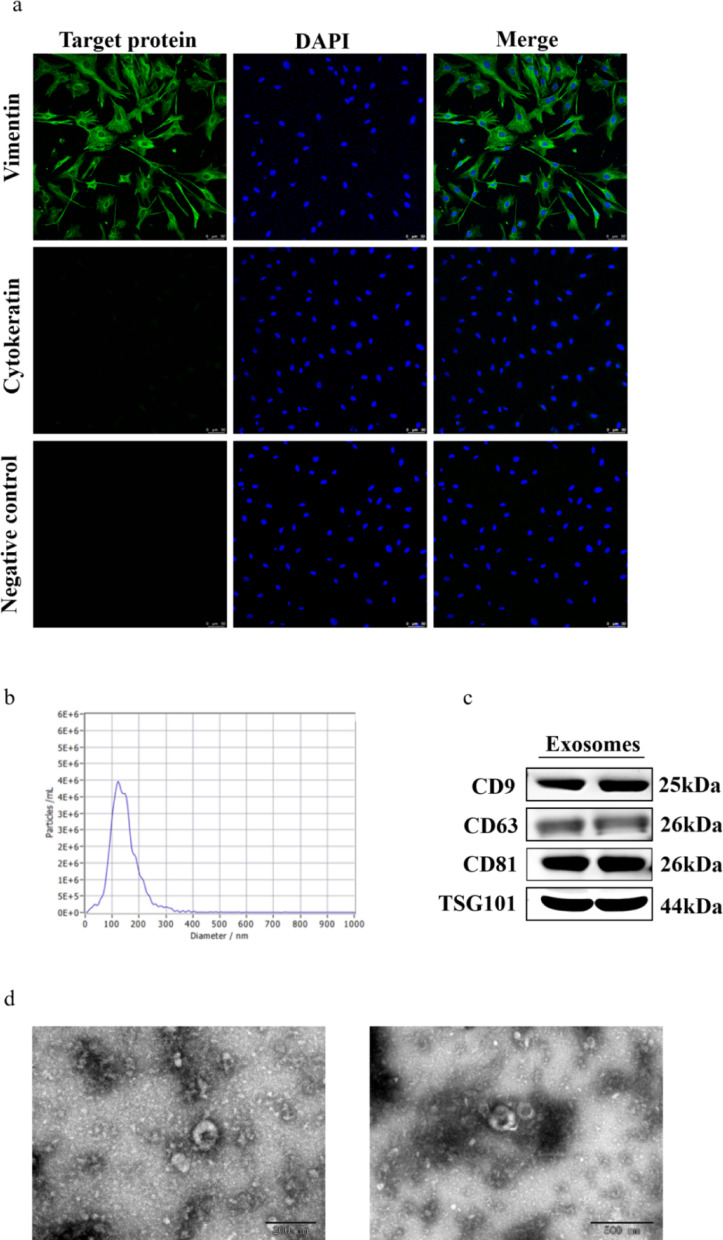
Characterization of exosomes and TCs in sheep follicles. **a** Immunofluorescence staining showed the expression of TCs marker Vimentin and epithelial cell marker Cytokeratin. Scale bars = 50 μm. **b** Size distribution of exosomes analyzed by Nanoparticle tracking analysis. **c** Western blot of exosomes marker CD9, CD63, CD81 and TSG101. **d** Electron microscope analysis of follicle fluid exosomes at different scales. Scale bars = 200, and 500 nm.

TCs were obtained by stripping membrane tissue from follicles 3–5 mm in diameter. Immunofluorescence staining showed that more than 95% of the cells expressed vimentin, and the cells were sufficiently pure and free of contamination to be used for subsequent experiments (Fig. 1a). The particle size of exosomes is distributed in the range of 30–150 nm, and the particle diameter of exosomes isolated in this experiment is mainly distributed at 124 nm, and the value of the particle diameter is consistent with the distribution range of the particle diameter of exosomes (Fig. 1b). Western blot results showed that exosome marker proteins (CD9, CD63, CD81 and TSG101) were expressed at high levels in the exosomes isolated in this experiment, indicating that the isolated exosomes were of high purity (Fig. 1c). The exosomes isolated in this experiment showed a cup-shaped structure, which has the general characteristics of exosome structure (Fig. 1d). Therefore, the integrity of the cells and exosomes isolated in this experiment is good to meet the requirements of subsequent experiments.

### Follicular fluid-derived exosomes promote TCs proliferation and steroid hormone synthesis

**Fig. 2:**
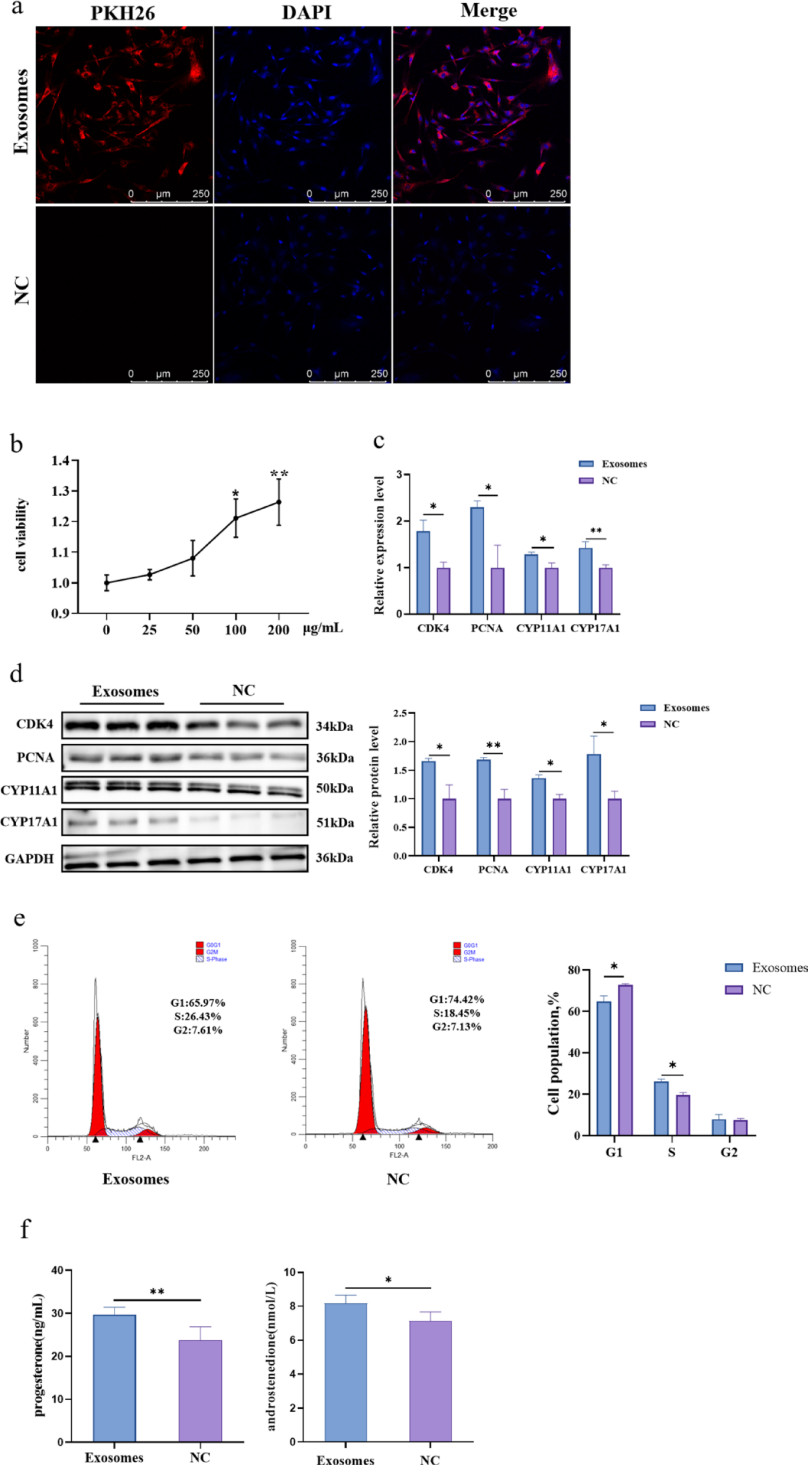
Exosomes promoted TCs proliferation and steroid hormone synthesis. **a** Result of TCs uptake follicle fluid exosomes. Red dots: Exosomes were stained with PKH26, blue dots: nucleus were stained with DAPI. Scale bars = 250 μm. **b** CCK-8 results of TCs treated with different concentrations of exosomes. **c** Detection of mRNA expression levels of *CDK4*, *PCNA CYP11A1* and *CYP17A1*. **d** The protein levels and gray values analysis were detected by western blot analysis. **e** Flow cytometric analysis was used to study the cell cycle of TCs. **f** Progesterone and androstenedione concentrations were measured by enzyme-linked immunosorbent assay. *N* = 3; *P < 0.05; **P < 0.01 *N* = 3

To assess the effect of exosomes on TCs development, treatment of TCs with PBS (NC) or exosomes was followed by PKH26 staining, which showed that exosomes labelled with red PKH26 fluorescence entered the TCs after 24 h of treatment and were evenly distributed in the cytoplasm (Fig. 2a). The activity of TCs was measured by the CCK-8 method by incubating with different concentrations of exosomes for 24 h. The results showed that exosomes enhanced the viability of TCs in a dose-dependent manner, with the highest viability observed in the 200 µg/mL group (Fig. 2b). We then treated the TCs with 200 µg/mL exosomes or PBS, and the RT-qPCR results showed that the mRNA expression levels of genes related to steroid hormone synthesis (*CYP11A1* and *CYP17A1*) and proliferation (*CDK4* and *PCNA*) were significantly higher in the exosomes-treated group than in the PBS group (Fig. 2c). This was in general agreement with the Western blot results (Fig. 2d). In addition, the number of S-phase cells in TCs was significantly increased in the exosomes group (Fig. 2e). ELISA results showed that the exosomes had higher levels of progesterone and androstenedione (Fig. 2f).

### Sequencing results and analysis

**Fig. 3:**
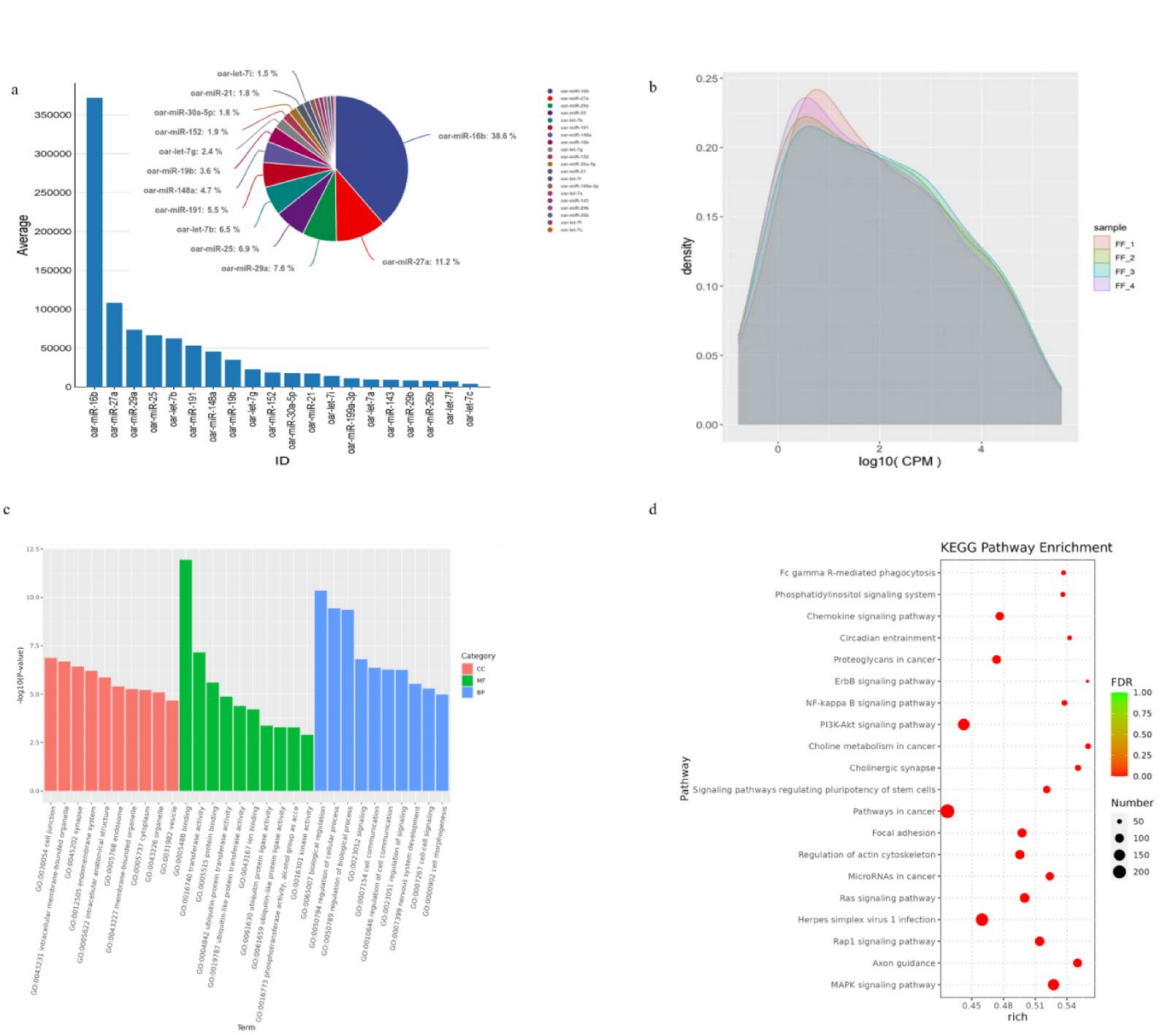
sRNA sequencing results and analysis. **a** Schematic of the TPM values of the top 20 miRNAs in the sequencing results, **b** TPM density distribution map, **c** GO analysis, **d** KEGG analysis, *N* = 4

To investigate the specific mechanisms by which exosomes affect membrane cells, we started with miRNAs, the most enriched component of exosomes, and identified a total of 130 miRNAs in follicular fluid exosomes by sRNA sequencing. The top 20 miRNAs with the highest expression levels were selected for further analysis (Fig. 3a). Analysis of the TPM densities showed that the distribution of gene expression was similar in the four samples, indicating that the samples were reproducible (Fig. 3b). A total of 37,343 target genes were predicted for the 20 miRNAs, and GO and KEGG analyses were performed on them. GO enrichment analysis revealed that biological regulation, regulation of cellular process and regulation of biological process were the most enriched biological processes. In terms of molecular functions, target genes of top 20 miRNAs were mainly enriched in binding, transferase activity and other aspects. In terms of cellular components, they were mainly enriched in intracellular membrane and synapse (Fig. 3c). The results of the KEGG enrichment analysis suggested that the target genes primarily regulate signalling pathways involving cancer, PI3K-AKT, MAPK, Rap1, Ras (Fig. 3d). These pathways are closely linked to follicular development and function.

### Effect of miR-148a in follicular fluid exosomes on TCs proliferation and steroid hormone synthesis

**Fig. 4:**
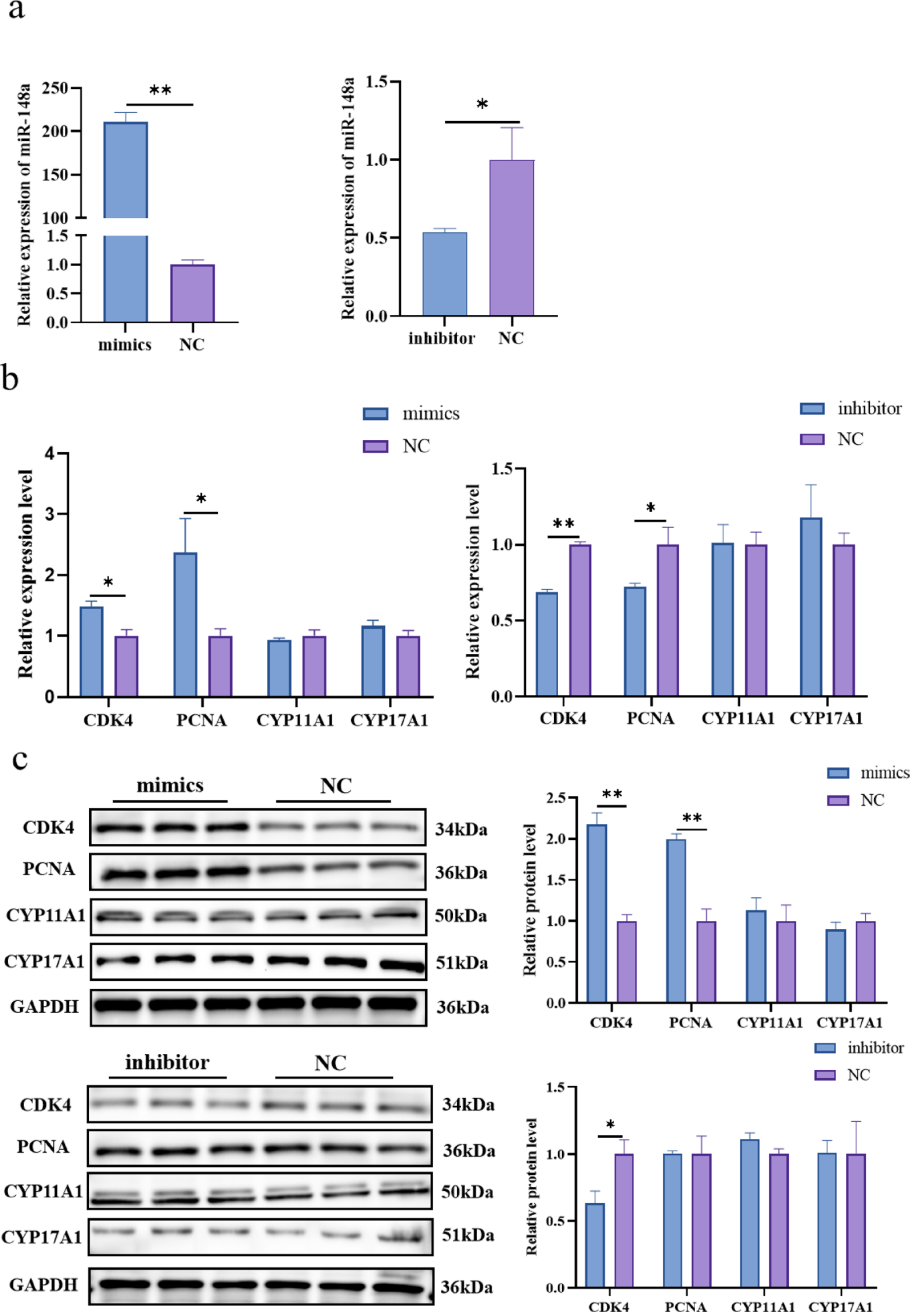

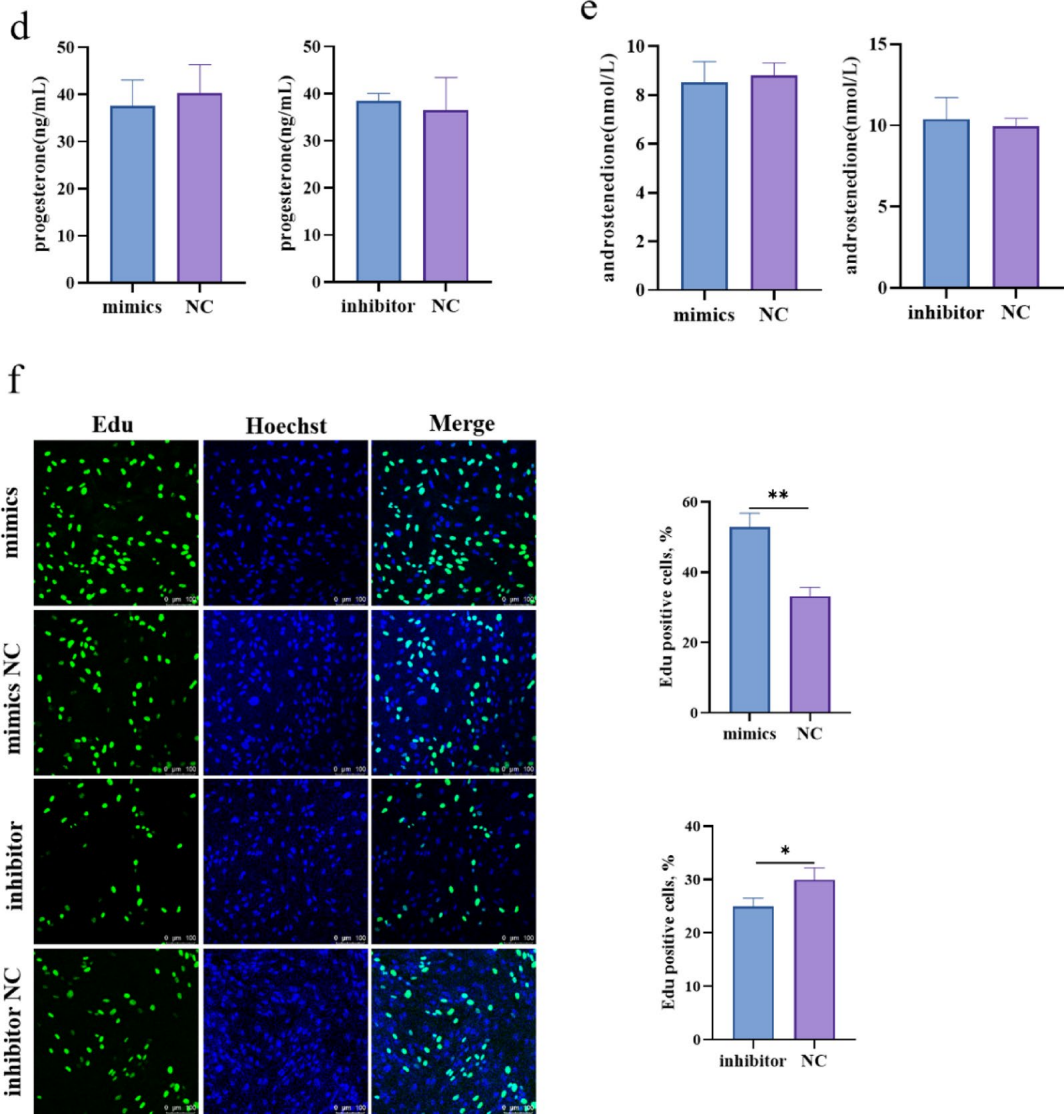
Effect of miR-148a on the proliferation and steroid hormone secretion of sheep TCs. **a** Overexpression and knockdown efficiency of miR-148a after transfection with miR-148a mimics and inhibitor. **b** Relative mRNA expression levels determine of CDK4, PCNA, CYP11A1 and CYP17A1 after miR-148a overexpression and knockdown. **c** Protein levels were detected by Western blot analysis after miR-148a overexpression and knockdown. **d** The concentration of progesterone was determined by enzyme-linked immunosorbent assay after miR-148a overexpression and knockdown. **e** The concentration of androstenedione was determined by enzyme-linked immunosorbent assay after miR-148a overexpression and knock-down. **f** EdU staining assays for the number of proliferation cell. Positive cells stained by EdU (green) and total cell nucleus stained with Hoechst (blue). **g** Flow cytometric analysis was used to study the cell cycle of TCs. N = 3; *P < 0.05; **P < 0.01 *N* = 3

Among the miRNAs highly expressed in follicular fluid exosomes, we found one miRNA of great interest. Previous studies in our laboratory have shown that miR-148a is differentially expressed in sheep with different productivity (unpublished data). Studies have reported that miR-148a plays an important role in follicular development [32]. Therefore, miR-148a was selected for further investigation. To determine the effect of miR-148a on TCs proliferation and hormone secretion in sheep, TCs were transfected with miR-148a mimics, inhibitors and negative controls. The expression of miR-148a was significantly increased and decreased after transfection with mimics and inhibitor, respectively (Fig. 4a). The mRNA and protein levels of cell proliferation-related genes (*CDK4* and *PCNA*) were significantly increased and decreased in the mimics and inhibitor groups, respectively, but neither of the steroid synthesis-related genes (*CYP11A1* and *CYP17A1*) changed significantly, although there was no significant change in the protein level of PCNA in the inhibitor group (Fig. 4b, c). ELISA results showed no significant changes in progesterone and androstenedione levels in either the mimics or inhibitor groups (Fig. 4d, e). The EdU staining assay showed an increase in the number of EdU-labelled positive cells in the mimic group and a decrease in the number of EdU-labelled positive cells in the inhibitor group compared to the control group (Fig. 4f). Flow cytometry analysis showed that mimics and inhibitors increased and decreased the percentage of S phase cells, respectively (Fig. 4g). In conclusion, miR-148a was found to promote the proliferation of TCs but did not affect steroid hormone secretion.

### MiR-148a specifically targets *TGFβ2* and inhibits its expression

**Fig. 5:**
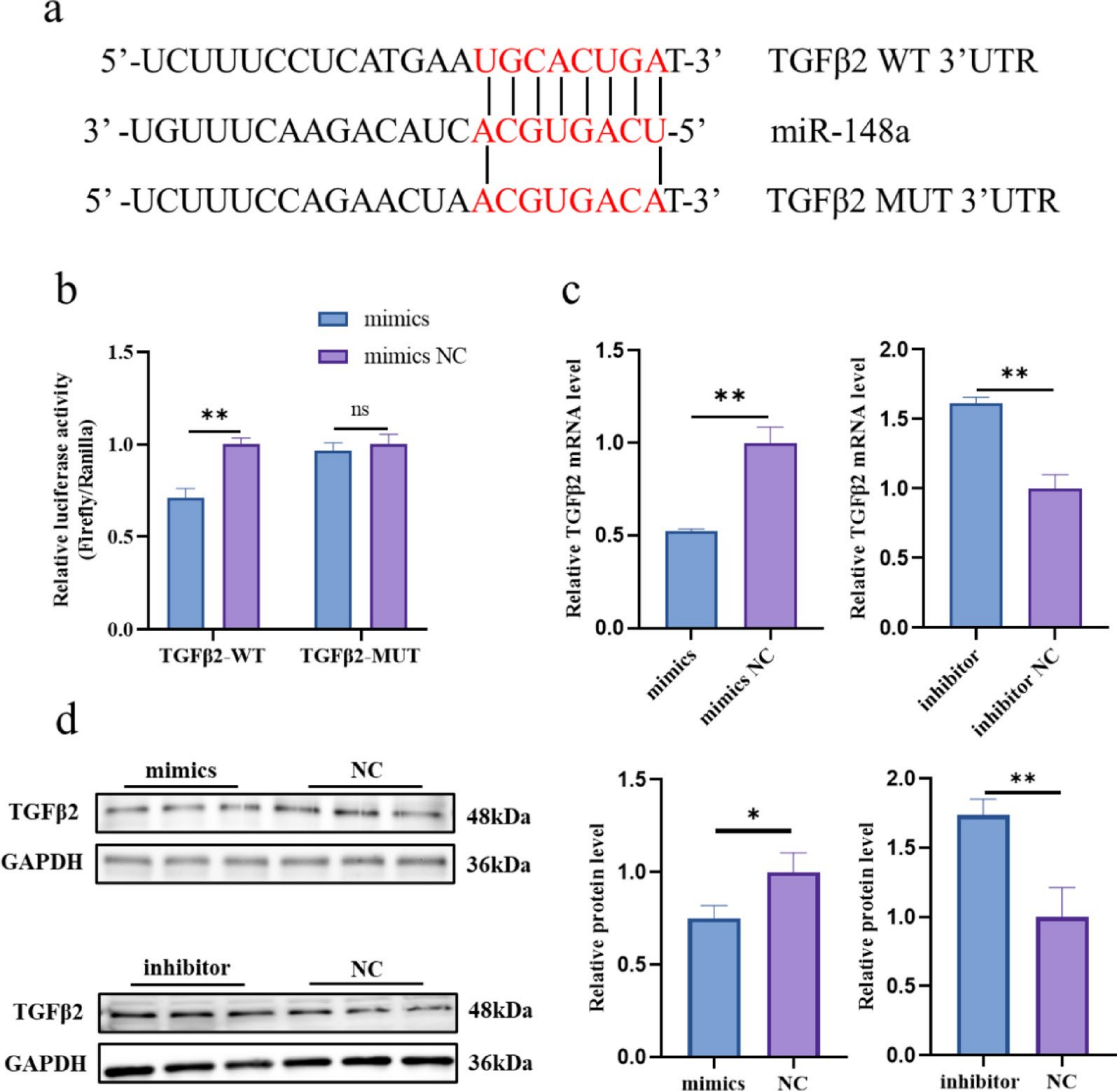
*TGFβ2* is one of the miR-148a target genes. **a** miR-148a binding site within the TGFβ2 3’UTR predicted. **b** Binding of miR-148a to the 3’-UTR of *TGFβ2* detected by dual luciferase activity. **c** Relative *TGFβ2* mRNA expression levels after treatment with miR-148a mimics or inhibitors. **d** Relative TGFβ2 protein expression levels after treatment with miR-148a mimics or inhibitors. *N* = 3; *P < 0.05; **P < 0.01; ns: no difference *N* = 3

To better understand the regulatory mechanisms of miR-148a in promoting the proliferation of TCs, the TargetScan8.0 and miRDB were used to predict its potential target genes. Combining site prediction and literature review, we identified TGFβ2 as a candidate target gene (Fig. 5a). Dual-luciferase reporter assays showed that miR-148a mimics significantly reduced the luciferase activity of TGFβ2 WT, while it had no significant effect on the luciferase activity of TGFβ2 MUT (Fig. 5b). RT-qPCR results showed that TGFβ2 mRNA level in sheep TCs was decreased after the miR-148a overexpression, and the opposite was true after inhibiting its expression (Fig. 5c). The results of the western blot analysis demonstrated a congruent trend between the protein and mRNA expression levels (Fig. 5d). Collectively, these findings indicate that TGFβ2 is a target gene of miR-148a.

### *TGFβ2* inhibits proliferation of sheep TCs

**Fig. 6:**
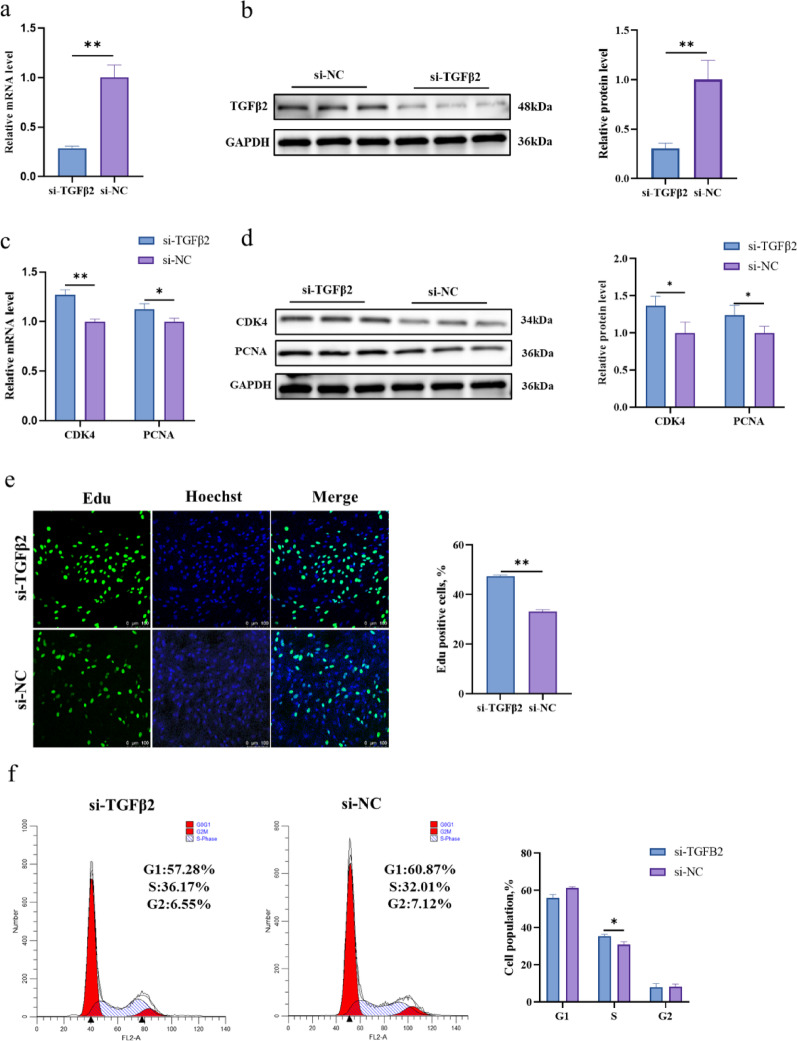
Inhibition of *TGFβ2* expression promoted TCs proliferation. **a** The interference efficiency of *TGFβ2* was measured using RT-qPCR. **b** Western blotting reveals the expression levels of TGFβ2. **c** RT-qPCR analysis of proliferation-related genes (*CDK4* and *PCNA*). **d** Protein levels were detected by Western blot analysis after TGFβ2 inhibition. **e** EdU staining assays proliferous cell quantities. Positive cells stained by EdU (green) and total cell nucleus stained with Hoechst (blue). **f** Flow cytometric analysis was used to study the cell cycle of TCs. *N* = 3; *P < 0.05; **P < 0.01 *N* = 3

To further verify the effect of TGFβ2 on TCs proliferation, siRNAs were designed and synthesized based on the TGFβ2 gene information and transferred into TCs together with siRNA-NC. The RT-qPCR results showed significant interference efficiency (Fig. 6a). And the Western blotting results are consistent with it (Fig. 6b). TGFβ2 inhibition increased the mRNA and protein expression levels of CDK4 and PCNA in the sheep TCs (Fig. 6c, d). Inhibition of TGFβ2 also increased the proportion of EdU-labelled positive cells (Fig. 6e). Flow cytometry showed that inhibition of TGFβ2 significantly increased the number of S-phase cells in TCs (Fig. 6f). These results suggested that TGFβ2 inhibited the proliferation of sheep TCs.

## Discussion

Recent studies show that follicular-fluid exosomes, many of which may originate from systemic plasma, act as key mediators of intercellular communication during follicular development [33]. However, the composition of follicular fluid exosomes is complex and their specific sources and regulatory mechanisms are not fully understood. In the present study, we successfully isolated follicular fluid exosomes from sheep and found that follicular fluid exosomes could be taken up by TCs and promoted TCs proliferation and steroid hormone secretion, which is consistent with previous findings [26]. Exosome-mediated miRNA transfer is one of the pathways for information transfer in organisms [34]. Therefore, we performed miRNA sequencing analysis on exosomes isolated from follicular fluid and found that the target genes of some miRNAs highly expressed in exosomes mainly regulate signalling pathways closely related to follicle development and function, such as PI3K-AKT, MAPK, Rap1 and Ras, which is similar to the results of other mammalian studies [35, 36].

It is interesting to note that among the miRNAs highly expressed in follicular fluid exosomes, we have identified miR-148a in transcriptome sequencing results. Recent studies have shown that miR-148a plays an important role in many physiological processes, includin lipid deposition and metabolism [37], milk quality [38], cell differentiation, proliferation and apoptosis [39]. In addition, miR-148a was shown to improve oocyte antioxidant capacity and promote oocyte development [35]. However, studies related to miR-148a regulation of TCs function in sheep have not been reported. In the present study, miR-148a was found to significantly increase cell proliferation in TCs, suggesting that miR-148a may affect follicular development by regulating cell proliferation in TCs. Target gene prediction, dual luciferase reporter gene assays, and RT-qPCR validation have confirmed that miR-148a directly targets the *TGFβ2* gene. TGFβ2 is an important member of the TGF-β family and is a secreted protein involved in many biological processes including cell growth, proliferation, migration and differentiation [40]. Ovarian somatic cells and oocytes express a wealth of growth factors, most belonging to the transforming growth factor-β (TGF-β) superfamily, in a developmentally stage-dependent manner, collectively forming the pivotal intra-ovarian network that orchestrates folliculogenesis [41]. TGFβ2 is present in follicular membrane cells at the beginning of the pre-sinusoidal developmental stage, as well as in the stromal tissue and vasculature of the ovary in sheep, but not in granulosa cells or oocytes [42]. Studies have shown that TGFβ2 induces apoptosis [43, 44]. In this study, we found that inhibition of TGFβ2 expression significantly reduced the proliferation of TCs in sheep.

By studying exosomes in sheep follicular fluid, we found that follicular fluid exosomes promote TCs proliferation and steroid hormone secretion, and that exosome-derived miR-148a promotes TCs proliferation by targeting TGFβ2 in them. Because steroid concentrations were reported per volume of medium and were not normalized to the number of viable cells, we cannot exclude the possibility that the higher progesterone and androstenedione levels in exosome-treated cultures are partly attributable to increased cell proliferation. Future experiments should quantify hormone output per cell (e.g., pg/10^4^ cells) to resolve this issue. In this study, we demonstrated that follicular fluid exosomes and their miR-148a regulate the function of sheep TCs, providing new insights into the possible exosome regulation of follicular development.

## Conclusions

In summary, sheep follicular fluid exosomes promoted TCs proliferation and hormone secretion. The mechanism may involve regulation of cell proliferation by miR-148a, which regulates the expression of *TGFβ2*. This process may improve the quality of follicle development, thereby promoting oocyte development.

## Data Availability

The datasets used and/or analyzed during the current study are available from the corresponding author on reasonable request.

## Abbreviations

CCK-8: Cell counting kit-8
CDK4: Cyclin-dependent kinase 4
CD9: CD9 molecule
CD63: CD63 molecule
CD81: CD81 molecule
CYP11A1: Cytochrome P450 family 11 subfamily A member
CYP17A1: Cytochrome P450 family 17 subfamily A member 1
EdU: 5-Ethynyl-20-deoxyuridine
ELISA: Enzyme-linked immunosorbent assay
GO: Gene ontology
KEGG: Kyoto encyclopedia of genes and genomes
MAPK: Mitogen-activated protein kinase
MUT: Mutant
NTA: Nanoparticle tracking analysis
PI3K-AKT: Phosphatidylinositol-3-kinase/protein kinase B
PCNA: Proliferating cell nuclear antigen
TCs: Theca cells
TEM: Transmission electron microscopy
TGFβ2: Transforming growth factor beta 2
TSG101: Tumor susceptibility 101
WT: Wild type
